# Acute and Long-Term Cardiovascular Complications among Patients with Sepsis and Septic Shock

**DOI:** 10.3390/jcm11247362

**Published:** 2022-12-12

**Authors:** Hamid Merdji, Martin Siegemund, Ferhat Meziani

**Affiliations:** 1 Faculté de Médecine, Université de Strasbourg (UNISTRA), Hôpitaux Universitaires de Strasbourg, Nouvel Hôpital Civil, Service de Médecine Intensive-Réanimation, 67000 Strasbourg, France; 2 INSERM (French National Institute of Health and Medical Research), UMR 1260, Regenerative Nanomedicine (RNM), FMTS, 67000 Strasbourg, France; 3 Intensive Care Unit, Department of Acute Medicine, University Hospital, 4056 Basel, Switzerland; 4 Department of Clinical Research, University of Basel, 4056 Basel, Switzerland

Sepsis is a life-threatening organ dysfunction caused by a dysregulated host response to infection and is the leading cause of death within intensive care units (ICUs). Resulting from a complex pathophysiological process characterized by an infection-induced generalized intravascular inflammatory state, sepsis and septic shock induce marked dysregulation of cardiovascular adaptive responses. Thus, sepsis has special links with the cardiovascular system, whether in the acute phase or longer term.

During the acute phase, vasoplegia is one of the main symptoms of septic shock. Its pathophysiology mainly involves endothelial dysfunction at the cardiovascular level and excessive production of vasodilating nitric oxide [1,2]. Exogenous pathogen-associated molecular patterns (PAMPs) and endogenous damage-associated molecular patterns (DAMPs) trigger activation of the endothelium via pathogen recognition receptors (PRRs) such as Toll-like receptors (TLRs). Endothelial structure and/or function is impaired by the reprogramming of endothelial cells toward a proinflammatory phenotype; this converts the protective monolayer that preserves physiological vascular tone to a pro-inflammatory, athero-thrombogenic profile with altered vasoregulation, a loss of barrier function, and coagulation activation, ultimately leading to microcirculatory disorders [3] and organ dysfunction. Sepsis management now has well-defined guidelines, such as those of the Surviving Sepsis Campaign [4]; they recommend the use of crystalloid solutions as first-line fluids for resuscitation for this state of relative hypovolemia, and norepinephrine as a first-line vasopressor agent for the treatment of vasoplegia in septic shock.

The cardiovascular system is also affected by autonomic nervous system dysfunction during sepsis and septic shock, generally referred to as autonomic dysfunction or dysautonomia [5]. Several studies have demonstrated dysfunction of the sympathetic branch of the autonomic nervous system in septic shock, with a maladaptive response to hypotensive and inflammatory stress; this leads to impaired autonomic control of the heart and vessels, contributing to circulatory failure and increased mortality. The pathophysiological mechanisms underlying this autonomic imbalance are not yet fully understood, but are known to include excessive, uncontrolled, or prolonged sympathetic nervous system activation, and/or inappropriate downregulation of the parasympathetic nervous system (the vagus nerve representing the main component of this system, also propagating the infection to regulatory brain centers). Thus, indirect cardiovascular indices of autonomic dysfunction include reduced heart rate variability and blood pressure variability, as well as attenuated baroreflex sensitivity.

Partly linked to dysautonomia, cardiac arrhythmias such as new-onset atrial fibrillation (AF) and ventricular arrhythmias can also affect septic patients. The most common of these arrhythmias is new-onset AF, which can affect up to 43% of ICU patients with varying severity of sepsis [6]. The underlying mechanisms leading to AF are still poorly understood, although proinflammatory cytokines, high levels of circulating stress hormones, autonomic dysfunction with a reduced parasympathetic regulatory effect, central venous catheter stimulation, intravascular volume shifts, and atrial macrophage and polymorphonuclear leukocyte infiltration may play a role.

Patients may also suffer ischemic events, among which myocardial infarctions (7.5%) seem to be more frequent than strokes (2.2%) [7]. Numerous studies have shown that the risk of ischemic events is associated with sepsis peaks at the onset of infection and is proportional to the severity of the illness [8]. Autopsy studies have shown increased inflammatory activity (including metalloproteinases and peptidases) in atheromatous plaques after an infectious stimulus, significantly contributing to plaque destabilization [8]. The prothrombotic, procoagulant state during sepsis increases neutrophil extracellular traps, platelet activity, and tissue factor (also known as coagulation factor III or thromboplastin) and impairs fibrinolysis, further increasing the risk of coronary thrombosis at sites of plaque disruption. 

Among myocardial infarctions, Type 2 myocardial infarction can result from a mismatch in myocardial oxygen demand and/or reduced supply in the absence of acute atherothrombotic plaque disruption [9]. During sepsis, inflammation and fever increase the metabolic demand of peripheral tissues and organs, resulting in tachycardia; this shortens the filling time during diastole, thereby mainly compromising left coronary perfusion. Hypoxia, arrhythmia, coronary microvascular dysfunction, and local toxin-mediated vasoconstriction may also contribute to this mismatch. In critically ill elderly patients, cardiac metabolic mismatch may also be increased by coronary stenosis from chronic plaques. Type 2 myocardial infarction is becoming more commonly diagnosed due to the increasing sensitivity of cardiac troponin assays and is also associated with higher short-term morbidity and mortality. However, once again, the optimal therapeutic regimen in clinical practice to treat this form of relative myocardial ischemia is still under debate.

The occurrence of new-onset cardiovascular events (including ischemic, acute heart failure, or AF) is not uncommon; according to [7], nearly 32% of patients hospitalized due to severe sepsis experienced at least one incidental cardiac event, which is not trivial, as it led to a 30% increase in the risk of death.

Cardiac dysfunction caused by sepsis, usually termed “sepsis-induced cardiomyopathy”, is also common; however, its prevalence estimation (between 22 and 60%) has varied widely, largely owing to the lack of a consensual objective definition to date [10]. Pathophysiology is complex and seems to involve cardiomyocytes, endothelial and mitochondrial dysfunction, and sympathetic hyperactivation. Although the extent to which this cardiovascular disorder affects outcomes remains unclear, data have recently been produced regarding patients who received mechanical circulatory support such as veno-arterial extracorporeal membrane oxygenation (VA-ECMO) for sepsis-induced cardiomyopathy with cardiogenic shock [11]. Takotsubo syndrome, mostly characterized by apical akinesis or ballooning with hyperdynamic basal segments and usually following an emotional or physical stressor, seems quite uncommon in sepsis [12].

At the crossroads between sepsis/septic shock and the heart is infective endocarditis. However, infective endocarditis is usually the primary etiology rather than a cardiovascular complication of sepsis/septic shock [13]. Nevertheless, with an aging population and the increasing use of implantable cardiac devices and heart valve replacements, it may become a more common cardiovascular complication of sepsis and septic shock in the future.

Similarly, myocarditis secondary to sepsis mainly occurs due to sepsis of viral origin, which has often been described in COVID-19 [14]; however, as with endocarditis, it is beyond the scope of this editorial.

Finally, regarding acute cardiovascular complications during sepsis, patients with a higher cardiovascular disease burden are at higher risk of death and of sudden cardiac arrest during sepsis and septic shock [15].

In the long term, among the consequences of sepsis (also termed “post-sepsis syndrome”), an increased risk of unexplained cardiovascular complications, such as myocardial infarction, stroke, acute heart failure, or AF, is one of the emerging specific health concerns [16,17,18]. Traditionally, large multi-center randomized controlled trials in critical illness have been focused on interventions aimed at decreasing early inpatient mortality, but the spotlight is increasingly turning to post-critical illness survivorship and quality of life. Indeed, recent data suggest that the increased risk of long-term mortality among sepsis survivors could be related to increased post-sepsis cardiovascular diseases. Recent data highlight sepsis as a possible long-term cardiovascular disease risk factor, with magnitudes of relative risk comparable to those of conventional risk factors such as hypertension, dyslipidemia, and diabetes mellitus. In [16], this increased risk of developing cardiovascular disease seemed to persist for at least 5 years and, interestingly, this also applies to the subgroup of sepsis survivors who did not have cardiovascular disease before hospitalization. The pathophysiology includes tissue remodeling in the vascular and cardiac beds through the activation of pro-fibrotic cellular signaling pathways, premature/accelerated vascular senescence, persistent derangements of lipid profile components, chronic inflammation, and inflammaging, leading to tissue fibrosis and atherogenesis [17,19,20]. Specific aspects of sepsis or septic shock that may predict long-term cardiovascular complications are under-studied; however, a recent study found an association between peak troponin I level (especially above 0.42 ng/mL) during sepsis and the subsequent risks of individual cardiovascular events in the year following sepsis [21].

The assessment and management of cardiovascular system pathophysiology are ongoing endeavors in intensive care units (ICUs), and given that most interventions have potential benefits and harms, additional data are critically important for evidence-based medicine. Interestingly, except for vasoplegia, there is no evidence or consensus on the management of most of these previously mentioned cardiovascular complications occurring during or after sepsis. Indeed, data that define optimal management strategies are limited for most of these acute and long-term cardiovascular complications in sepsis. Studies are urgently required not only to improve survival, but also to reduce morbidity, prevent organ failure, and shorten the convalescence of patients experiencing sepsis and septic shock.
